# The Protective Effect of Liquiritin in Hypoxia/Reoxygenation-Induced Disruption on Blood Brain Barrier

**DOI:** 10.3389/fphar.2021.671783

**Published:** 2021-07-06

**Authors:** Mengting Li, Jia Ke, Yiqing Deng, Chunxiang Chen, Yichen Huang, Yuefeng Bian, Shufen Guo, Yang Wu, Hong Zhang, Mingyuan Liu, Yan Han

**Affiliations:** ^1^ Department of Neurology, Yueyang Hospital of Integrated Traditional Chinese and Western Medicine, Shanghai University of Traditional Chinese Medicine, Shanghai, China; ^2^ Institute of Interdisciplinary Integrative Biomedicine Research, Shanghai University of Traditional Chinese Medicine, Shanghai, China

**Keywords:** liquiritin, vascular protection, human brain microvascular endothelial cells, blood-brain barrier, oxidative stress, endoplasmic reticulum stress

## Abstract

**Background:** Stroke is the second leading cause of death in human life health, but current treatment strategies are limited to thrombolytic therapy, and because of the tight time window, many contraindications, and only a very small number of people can benefit from it, new therapeutic strategies are needed to solve this problem. As a physical barrier between the central nervous system and blood, the blood-brain barrier (BBB) maintains the homeostasis of the central nervous system. Maintaining the integrity of the BBB may emerge as a new therapeutic strategy. Liquiritin (LQ) is a flavonoid isolated from the medicinal plant *Glycyrrhiza uralensis* Fisch. ex DC. (Fabaceae), and this study aims to investigate the protective effects of LQ on brain microvascular endothelial cells (BMECs), to provide a new therapeutic strategy for stroke treatment, and also to provide research ideas for the development of traditional Chinese medicine (TCM).

**Methods:** The protective effects of LQ on HBMECs under the treatment of hypoxia reoxygenation (H/R) were investigated from different aspects by establishing a model of H/R injury to mimic ischemia-reperfusion *in vivo* while administrating different concentrations of LQ, which includes: cell proliferation, migration, angiogenesis, mitochondrial membrane potential as well as apoptosis. Meanwhile, the mechanism of LQ to protect the integrity of BBB by antioxidation and inhibiting endoplasmic reticulum (ER) stress was also investigated. Finally, to search for possible targets of LQ, a proteomic analysis approach was employed.

**Results:** LQ can promote cell proliferation, migration as well as angiogenesis and reduce mitochondrial membrane potential damage and apoptosis. Meanwhile, LQ can also reduce the expression of related adhesion molecules, and decrease the production of reactive oxygen species. In terms of mechanism study, we demonstrated that LQ could activate Keap1/Nrf2 antioxidant pathway, inhibit ER stress, and maintain the integrity of BBB. Through differential protein analysis, 5 disease associated proteins were found.

**Conclusions:** Studies have shown that LQ can promote cell proliferation, migration as well as angiogenesis, and reduce cell apoptosis, which may be related to its inhibition of oxidative and ER stress, and then maintain the integrity of BBB. Given that five differential proteins were found by protein analysis, future studies will revolve around the five differential proteins.

## Introduction

With the increase of human life span and the aging of population, the incidence rate of age-related cerebrovascular diseases such as stroke has increased dramatically (GBD 2016 Lifetime Risk of Stroke Collaborators, 2018). According to the Global Burden of Diseases (GBD), the age-standardized mortality rate of cerebral infarction is 56.9/100000, which is the second leading cause of death in the world and the first cause of death in China (GBD 2017 Causes of Death Collaborators, 2018). At present, the most effective method for ischemic brain injury is limited to vascular recanalization, and the recovery of blood flow will increase the reperfusion injury (Powers et al., 2019). In addition, high time window and many contraindications lead to less than 20% of patients can benefit from thrombolysis (Molina, 2011; Widimsky et al., 2014). Therefore, there is an urgent need for new treatment strategies and drugs to expand the beneficiaries.

After the occurrence of ischemic stroke, blood-brain barrier (BBB), the physical barrier between the central nervous system (CNS) and blood, is destroyed, which accelerates the damage process of brain parenchyma. Under physiological conditions, BBB protects brain tissue from inflammatory and toxic substances, and maintains the stability of CNS (Abbott et al., 2010; Keep et al., 2014). Brain microvascular endothelial cells (BMECs), pericytes and astrocytes jointly maintain the integrity of BBB. Ischemia reperfusion can mediate BBB rupture, resulting in edema and brain parenchymal damage (Granger and Kvietys, 2015; Keaney and Campbell, 2015). It has been reported that BMECs injury plays an important role in ischemia-reperfusion injury after cerebral infarction (Wu et al., 2019). Therefore, protecting BMECs may be a potential therapeutic strategy for stroke treatment.

With a history of more than 2000 years, Chinese herbs is an important part of traditional Chinese medicine (TCM) (Hao et al., 2015). Although TCM is used as a supplementary and alternative medicine in many developed countries, in China, it’s applied to prevent and treat diverse diseases by more than 70% Chinese (Chen and Lu, 2006). In recent years, with the development of TCM, it is accepted by more and more countries, such as the United States and Australia (Guo et al., 2013). According to the Pharmacopoeia of the People’s Republic of China, *Glycyrrhiza uralensis* has the effects of tonifying spleen and Qi, clearing away heat and detoxification. Liquiritin (LQ), the main active component of *Glycyrrhiza uralensis* Fisch. ex DC. (Fabaceae), has anti-inflammatory and anti-oxidation effects, and can enter the brain tissue through BBB after ischemia-reperfusion injury (Li et al., 2015). However, whether it can exert a cerebroprotective effect remains unknown.

In our study, we discussed the protective effect of LQ on HBMECs injured by hypoxia reoxygenation (H/R), and studied the possible mechanism of LQ, so as to provide more theoretical basis for the clinical promotion of LQ and provide new ideas for the treatment of stroke.

## Materials and Methods

### Media, Reagents and Antibodies

Liquiritin (purity > 98%) was acquired from DingRich Chemical Co., Ltd (Shanghai, China). HBMECs were bought from Qingqi Biotechnology Development Co., Ltd (Shanghai, China). Fetal bovine serum (FBS) was purchased from Biological Industries (Israel). Penicillin/streptomycin, phosphate buffer saline (PBS) and dulbecco's modified eagle medium (DMEM) were obtained from Hyclone (United States). Cell Counting Kit-8 (CCK-8) was purchased from Dongren Chemical Technology (Shanghai, China). Matrigel was bought from Corning (New York, United States). Mitochondrial membrane potential (JC-1) test kit, BCA protein test kit and NP40 lysate were purchased from Beyotime Biotechnology (Shanghai, China). Apoptosis Kit was purchased from Becton, Dickinson and Company (New York, United States). Human Superoxide dismutase (SOD) ELISA kit was obtained from Novus (United States). Malondialdehyde (MDA) ELISA kit was purchased from Biovision (San Francisco, United States). Human intercellular cell adhesion molecule-1 (ICAM-1) ELISA kit, human vascular cell adhesion molecule-1 (VCAM-1) ELISA kit and anti-Claudin-5 antibody were purchased from Abcam company (Cambridge, UK). GAPDH antibody was purchased from Affinity (United States). Nuclear factor E2-related factor 2 (Nrf2), Kelch Like ECH Associated Protein 1 (Keap1), ATF6, glucose-regulated protein 78 (GRP78), zonula occludens 1 (ZO-1) antibodies were purchased from Cell Signaling Technology (Danvers, United States). Horseradish peroxidase (HRP)-conjugated anti-rabbit IgG was brought from Jackson company (Pennsylvania, United States). SDS-PAGE rapid dispensing kit and ECL chromogenic solution were obtained from EpiZyme Biotechnology (Shanghai, China). TBST, electrophoretic buffer and electrotransfer solution were purchased from Beijing Solarbio Technology (Beijing, China).

### Cell Culture and H/R Treatment

HBMECs were cultured in high glucose medium containing 10% FBS, 1% streptomycin and penicillin, and were cultured in 5% CO_2_ at 37°C incubator. To simulate ischemia-reperfusion injury *in vivo*, the cell injury model of HBMECs was described previously with slight modifications (Liberale et al., 2020). The specific operation is as follows: HBMECs were firstly cultured in the basic medium without FBS under hypoxic conditions (1% O_2_, 5% CO_2_ and 94% N_2_) for 12 h. The medium then replaced by the complete medium containing FBS, and cultured under normal conditions (5% CO_2_ and 95% air) for 8 h.

### The Treatment of LQ

LQ was dissolved by DMSO at a stock concentration of 1 mmol/L. Then diluted to different concentrations with medium before use. The model group and the normal group were given medium containing 0.1% DMSO.

### Cell Viability Assay

HBMECs were seeded in 96-well plates with 5 × 10^3^ cells/well and incubated in complete medium under normal oxygen incubator overnight. To explore the safety dose of LQ, cells were incubated in the presence of 0.1, 0.5, 1, 2, 5, 10 μmol/L for 24 h in serum free medium under normal incubators. Then the safe dose was chosen for the next experiment. Cells were incubated in the presence of LQ (0.1, 0.5, 1 μmol/L) for 12 h in serum free medium under hypoxic incubators. Cells were then incubated with the same concentration of LQ as above for 8 h in complete medium under normal condition. Cell viability was determined by CCK-8 method according to the manufacturer’s instructions. Six holes were designed and repeated three times.

### Scratch Healing Assay

HBMECs were seeded in a 12-well plate with 2 × 10^5^ cells/mL and incubated in normal condition. Since the cells were covered in the holes, a straight line was drawn along the bottom of the plate with a 10 μL gunhead and photographed immediately (0 h). After the scratched cells were washed with PBS, different concentrations of LQ were added, and after H/R treatment, photographs were taken again. ImageJ was used to calculate the size of the damaged area enclosed by cells photographed twice. The final expression was the area relative to the control group. Two duplicate holes were set for each concentration and repeated three times.

### Tube Formation Assay

Matrigel (9–12 mg/ml) was plated in a 96-well plate with 50 μL/well and allowed to curdle at 37°C in normal incubator for 30 min. HBMECs were seeded in a 24-well plate with 2 × 10^5^ cells/mL and incubated in normal condition overnight. Different concentrations of LQ were added and the cells were digested and collected after the treatment of H/R. HBMECs were placed 5 × 10^5^ cells/mL per well on the pre-solidified matrigel, then cultured in a 37°C normal incubator for 8 h. The tube formation was observed under the microscope, and take pictures, five pieces per well. The length and number of the branch of the tube was calculated by software, which was finally expressed as the length relative to the control group. Two multiple holes were set for each concentration, and the experiment was repeated three times.

### Mitochondrial Membrane Potentials Assay

HBMECs were seeded in 12-well plates with 5 × 10^4^ cells/mL and incubated in 37°C, containing 5%CO_2_ incubator overnight. After treatment of different concentrations of LQ under H/R condition, JC-1 probe was employed to measure the change of mitochondrial membrane potentials. Briefly, 500 μL 1 × JC-1 staining working fluid was added into each well, incubated in the incubator for 20 min, washed twice with 1 × JC-1 buffer, and finally 300 μL PBS was added to take pictures with high content cell analyzer, and the fluorescence intensity was calculated with ImageJ software. Two multiple wells were set for each concentration and the experiment was repeated three times.

### Apoptosis Assay

Annexin V and PI staining were employed to measure apoptosis detection. HBMECs were treated with different concentrations of LQ and incubated in H/R condition. Then cells were digested and collected, after centrifuged and washed with pre-cooled PBS, 1 × binding buffer was used to resuspended. The fluorescein-conjugated Annexin Ⅴ and PI reagent were added to cell suspensions. Then the suspensions were incubated in dark for 10 min and were assessed by Beckman flow cytometer. The experiment was repeated three times.

### Enzyme-Linked Immunosorbent Assay (ELISA)

After treatment of HBMECs with LQ and H/R, the supernatants were collected. The expression of ICAM-1, VCAM-1, SOD and MDA were detected by ELISA according to the manufacturer's protocol.

### Reactive Oxygen Species (ROS) Assay

HBMECs were treated with different concentrations of LQ and H/R, DCFH-DA probe was loaded into cells, which can be oxidized by ROS to generate fluorescence and can be detected. After incubating at 37°C for 30 min, the probes that did not enter the cells were fully removed by washing them three times with serum-free medium. Finally, the high content cell analyzer was used to take pictures, and the fluorescence intensity was calculated by software with the control group as the reference. The experiment was repeated at least three times.

### Western Blot Assay

HBMECs were treated with LQ (0.1, 0.5, 1 μmol/L) and H/R, then the cells were collected for subsequent experiments. BCA kit was employed to measure the protein concentration, and 30 μg proteins were used in isolation of SDS-PAGE. After the proteins were transferred to polyvinylidene difluoride (PVDF) membrane, 5% of skimmed milk powder is used to block making the binding of the first and second antibodies. Membranes were then incubated with antibodies against Nrf2 (Rabbit, 1:1,000), Keap1 (Rabbit, 1:1,000), ATF6 (Rabbit, 1:1,000), GRP78 (Rabbit, 1:1,000), ZO-1 (Rabbit, 1:1,000), Claudin-5 (Rabbit, 1:1000) 16–18 h at 4°C. Subsequently, the first antibodies were adsorbed and membranes were washed with Tris Buffered saline Tween (TBST) for three times, horseradish peroxidase conjugated goat-anti-rabbit secondary antibodies (IgG-HRP, 1:10,000) were incubated within 1 h. Finally, the enhanced chemiluminescent (ECL) reagents were used for development. The expression of GAPDH (Rabbit, 1:10,000) was used as an internal reference. The experiment was repeated at least three times.

### Proteomic Analysis

HBMECs were treated with LQ of 1 μmol/L and H/R, then the cells were collected. The cells were gently scraped off from the culture plate, quickly put into liquid nitrogen for quick freezing, and then moved to −80°C for storage. The proteins extracted from cells were quantified by iTRAQ labeling, and the proteins with *p* value < 0.05 and ratio multiple change >1.2 or <0.83 were defined as differentially expressed proteins. Then, these differentially expressed proteins were analyzed by GO functional enrichment, and the related differential proteins were found.

### Statistical Analysis

The GraphPad Prism 8 was used for statistical analysis. Two independent samples were analyzed by *t*-test, and the measurement data was described by mean ± Standard deviation (SD). The comparison between groups was conducted by one-way analysis of variance (ANOVA). The *p* value less than 0.05 indicated that the difference was statistically significant.

## Results

### LQ can Enhance Cell Viability

**FIGURE 1:**
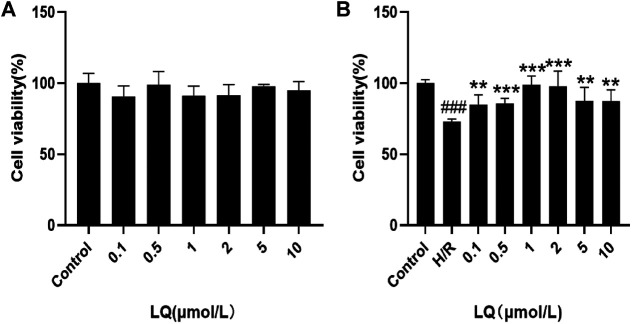
LQ increase cell vitality. **(A)** LQ under the concentrations of 0.1–10 μmol/L had no effect on HBMECs. **(B)** The ability of H/R-HBMECs could be increased under various concentrations of LQ (0.1, 0.5, 1 μmol/L). Every experiment was performed less than three times. ^###^ *p* < 0.001, compared to control. **p < 0.01, ***p < 0.001, compared to H/R.

To detect the effect of LQ on cell viability, CCK-8 kit was used. First, the safe dose of the drug was detected. As shown in Figure 1A, LQ had no effect on cell viability at 0.1–10 μmol/L. Subsequently, in order to find the effective dose of LQ, H/R-HBMECs were established. As shown in Figure 1B, LQ could promote the cell viability of H/R-HBMECs at 0.1–1 μmol/L. Ultimately, our study demonstrated that LQ could enhance cell viability in a certain concentration range.

### LQ Promotes Migration and Tube Formation of H/R-HBMECs

**FIGURE 2:**
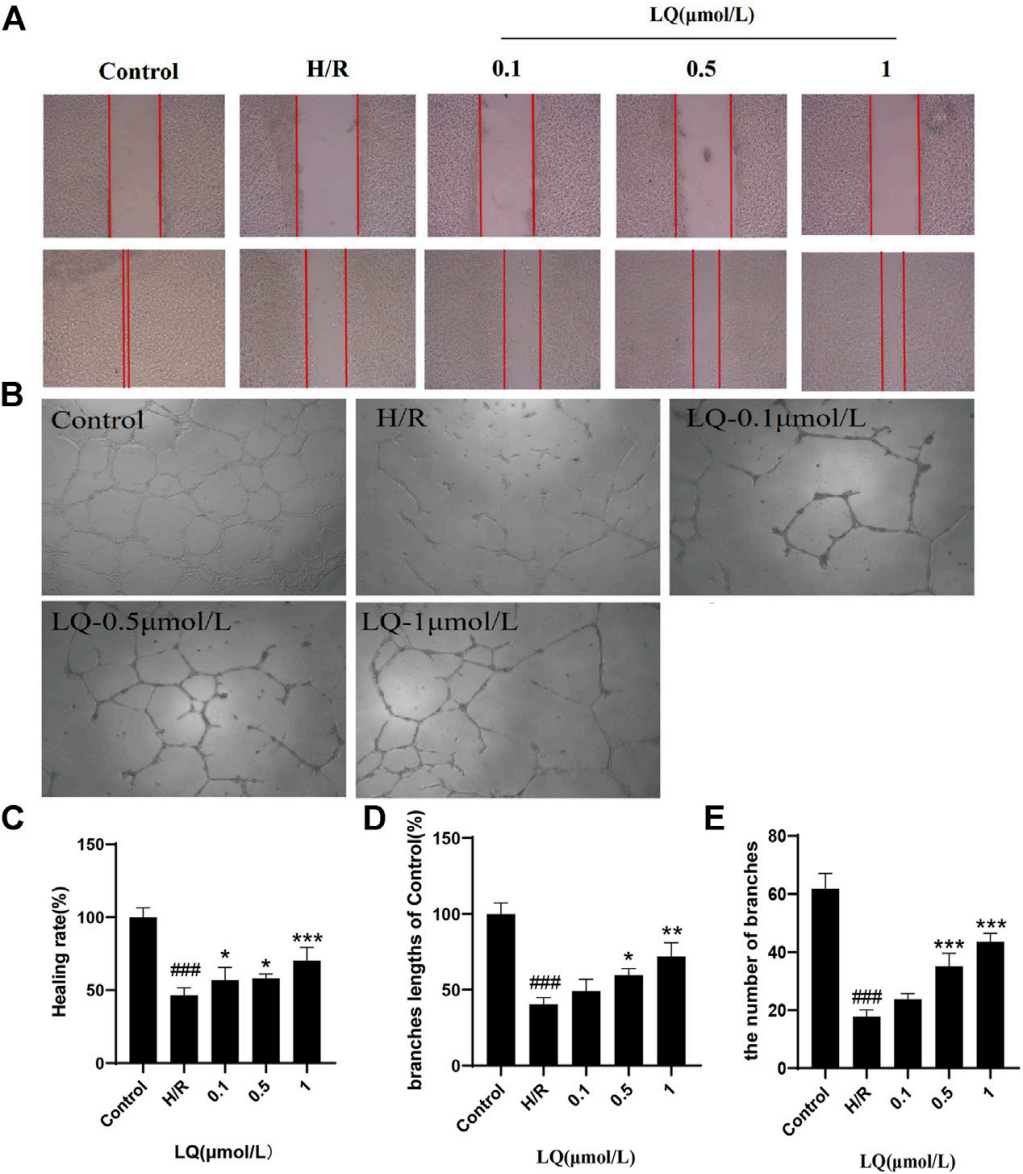
LQ promote migration and tube formation of H/R-HBMECs. **(A)** The distance between scratches could be reduced in the presence of LQ (0.1, 0.5, 1 μmol/L). **(B)** The branches length of the tube could be increased by different concentrations of LQ (0.5, 1 μmol/L). **(C)** The healing rate data analyze. **(D)** The branches length of the tube dada analyze. **(E)** The number of branches of tube data analyze. ^###^ *p* < 0.001, compared to control. *p < 0.05, **p < 0.01, ***p < 0.001, compared to H/R.

To assess the migration capacity of LQ on H/R-HBMECs, wound healing assay was examined for healing rate. As shown in Figures 2A,C, after the treatment of H/R, the migration capacity of HBMECs reduced, while LQ could reverse this phenomenon dose-dependently. Moreover, to test the effects of LQ on tube formation of HBMECs under the treatment of H/R, Matrigel assay was used to simulate the angiogenesis experiment. Robust and complete tubular-like structures of HBMECs were observed in normal group, while tube formation was disrupted by H/R. However, vessel branch length and number could be increased in a dose-dependent manner under LQ treatment (Figures 2B,D,E). Our results indicate that LQ can promote cell migration and angiogenesis in a dose-dependent manner.

### LQ can Reduce Apoptosis

**FIGURE 3:**
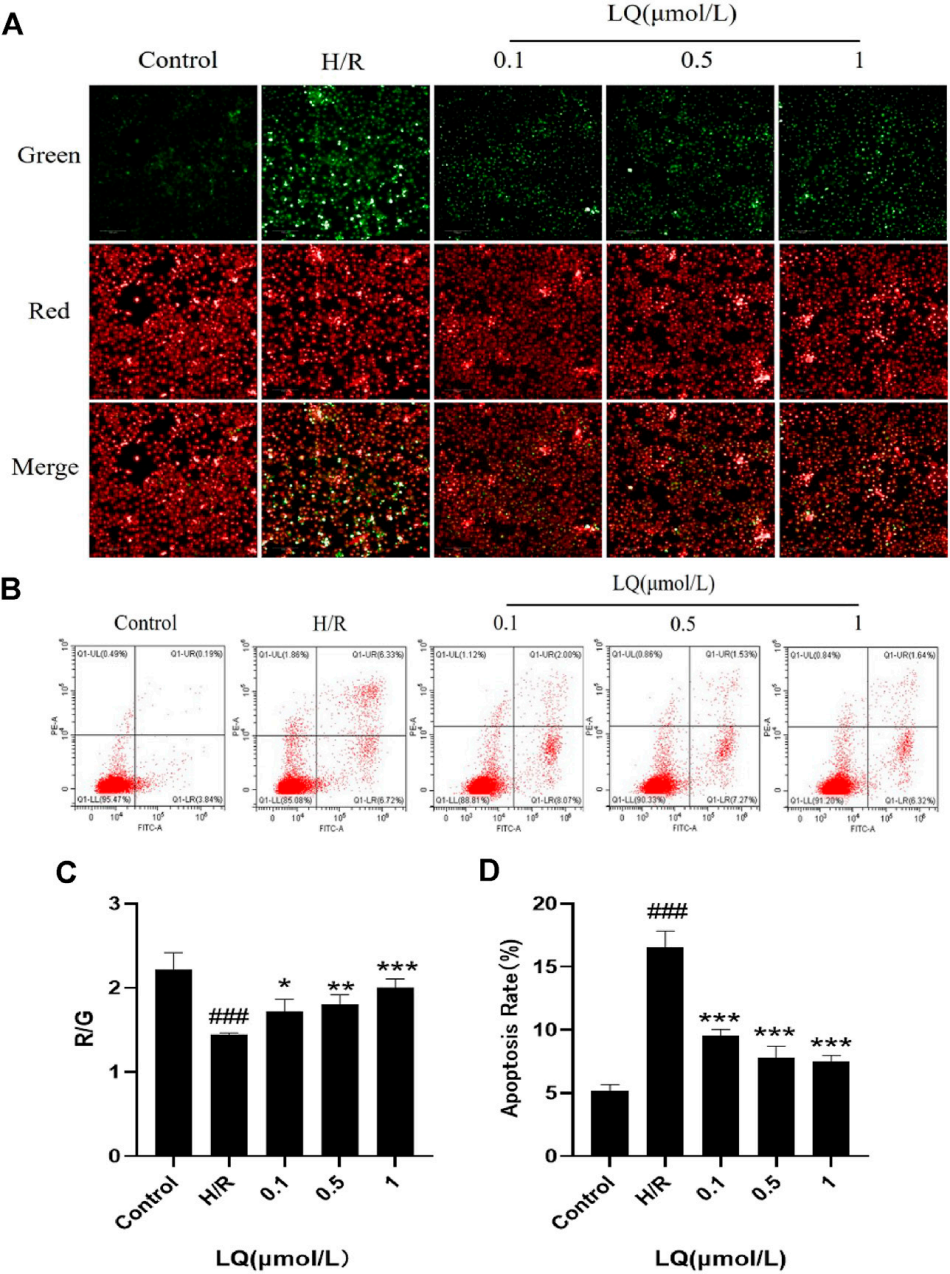
LQ can reduce apoptosis. **(A, C)** The mitochondrial membrane was increased by LQ. Green represents the decrease of membrane potential and red was increased. The results are expressed as the intensity of red fluorescence relative to green fluorescence. **(B, D)** The upper left corner represents necrotic cells due to mechanical injury, the upper right corner represents late apoptotic cells, the lower left corner represents normal cells, and the lower right corner represents early apoptosis. Flow cytometry detection of LQ can reduce cell apoptosis. ^###^ *p* < 0.001, compared to control. *p < 0.05, **p < 0.01, ***p < 0.001, compared to H/R.

It is well known that mitochondrial membrane potential decreases when cells are suffered from early apoptosis. To detect early apoptosis, JC-1 probe was used to detect changes in mitochondrial membrane potential. As shown in Figures 3A,C, the green fluorescence intensity was higher in the H/R group, which indicated that the membrane potential decreased and the cells underwent early apoptosis. However, in the presence of LQ, red fluorescence is relatively more, showing that early apoptosis is reduced. In order to further assess apoptosis, flow cytometry can be more intuitive detection. As shown in the Figures 3B,D, the apoptotic rate was significantly increased in the H/R group compared with the normal group. LQ can reduce cell apoptosis in a concentration-dependent manner. Our results showed that LQ could reduce apoptosis in a certain concentration range.

### LQ Reduces the Elevation of ICAM-1 and VCAM-1

**FIGURE 4:**
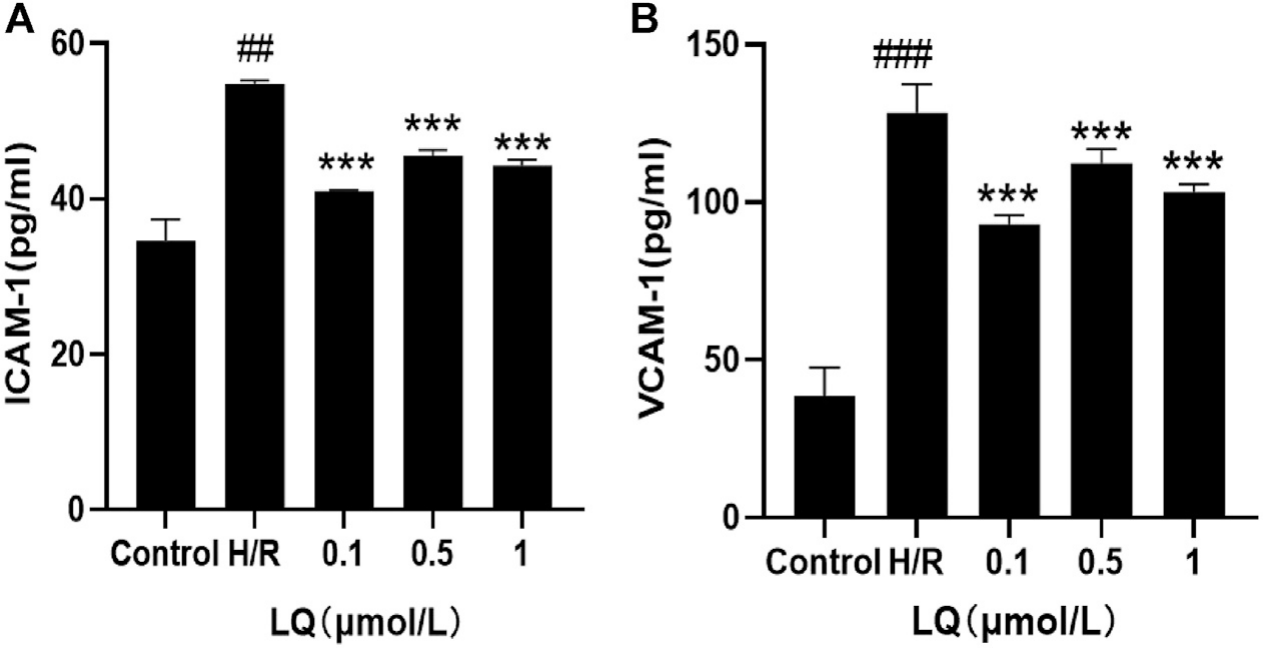
LQ decrease the increase of ICAM-1 and VCAM-1. **(A)** Compared with control, the expression of ICAM-1 was increased in H/R group, and LQ could reduce the elevated ICAM-1. **(B)** Compared with control, the expression of VCAM-1 was increased in H/R group, and LQ could reduce the elevated VCAM-1. ^##^ *p* < 0.01, ^###^ *p* < 0.001, compared to control. ***p < 0.001, compared to H/R.

In order to detect the effect of LQ on the surface adhesion of HBMECs, the levels of ICAM-1 and VCAM-1 were detected by ELISA kit, which are important adhesion molecule mediating the adhesion reaction. As shown in Figure 4, LQ can alleviate the elevation of ICAM-1 and VCAM-1 caused by H/R, thereby reducing cell-to-cell or cell-to-matrix adhesion, reducing endothelial cell permeability, and further ensuring the integrity of the BBB.

### LQ Regulates ROS, SOD and MDA Levels

**FIGURE 5:**
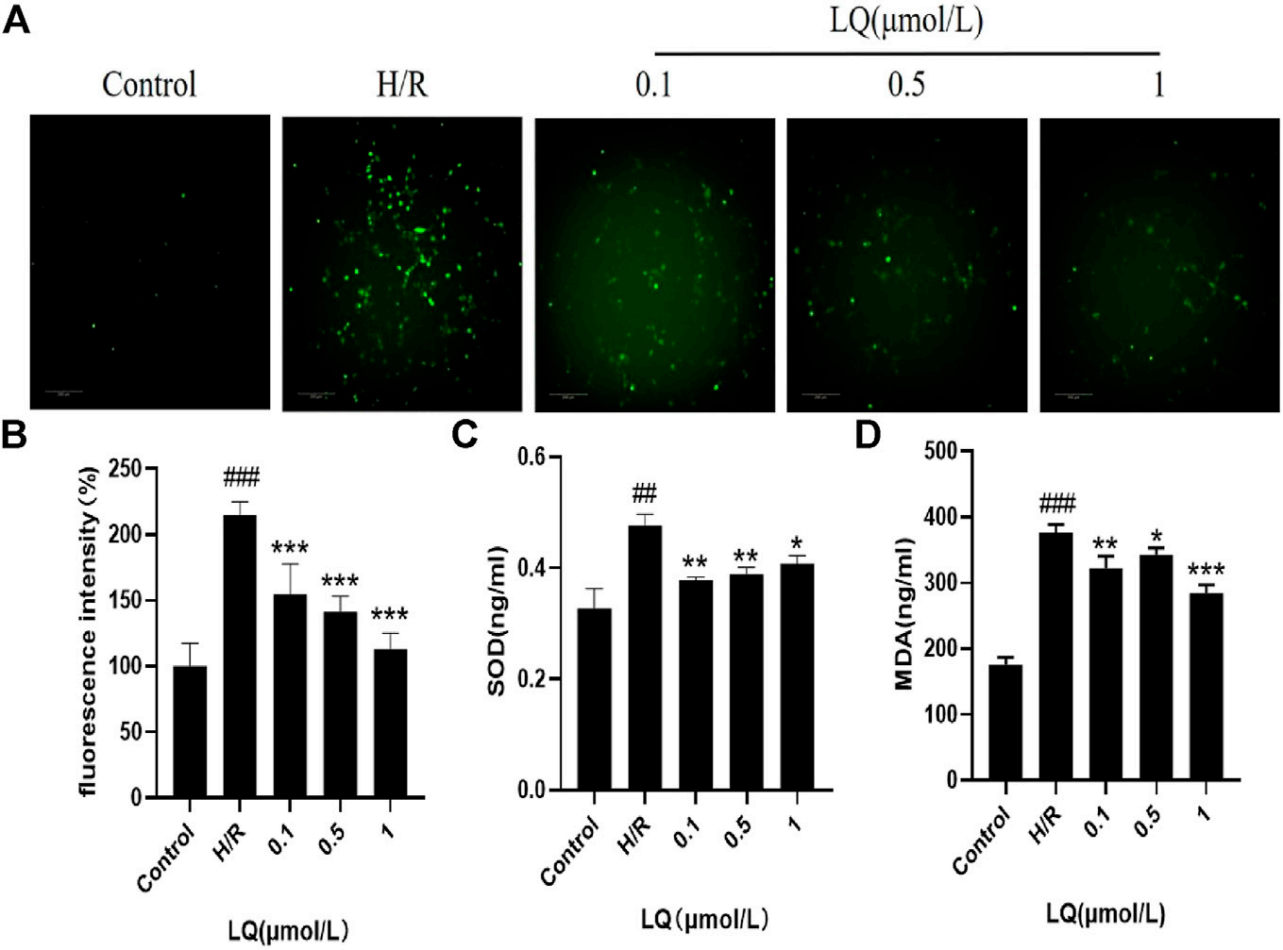
LQ regulates ROS, SOD and MDA levels. **(A, B)** Green fluorescence represents ROS, compared with control, green fluorescence intensity in H/R group is enhanced, representing the increase of ROS level, and LQ can reduce ROS level. **(C)** SOD level was increased by H/R, and LQ reduced SOD level. **(D)** MDA level was increased by H/R, and LQ decreased MDA level. ^##^ *p* < 0.01, ^###^ *p* < 0.001, compared to control. *p < 0.05, **p < 0.01, ***p < 0.001, compared to H/R.

Since oxidative stress can occur in ischemic events, we explored whether LQ can reduce oxidative stress caused by H/R. High content cell analysis system was used to detect the fluorescence intensity of ROS. In our study, green fluorescence was enhanced in the model group compared with the control group, indicating that oxidative stress events occurred. As shown in Figure 5A, LQ at a concentration of 0.1–1 μmol/L can reduce the production of ROS, then reduce oxidative stress, and protect cells from damage. Meanwhile, the detection of cell supernatant by ELISA indicated, SOD and MDA level increased in H/R group, as shown in Figures 5B,C. However, LQ could significantly attenuate the above function of HBMECs.

### LQ Regulates Oxidative Stress and Endoplasmic Reticulum (ER) Stress Pathway Proteins to Maintain BBB Integrity

**FIGURE 6:**
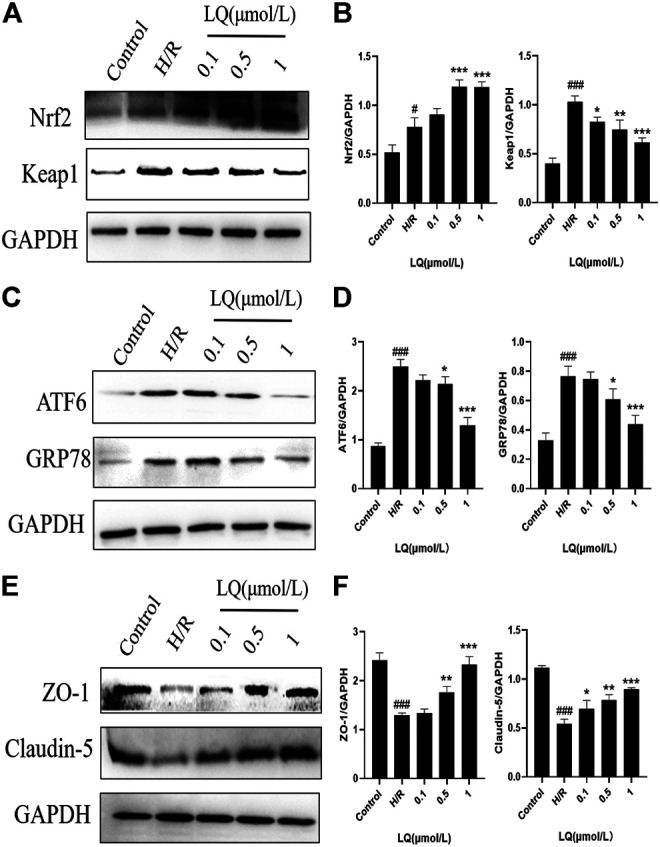
LQ regulations oxidative stress and ER stress pathway proteins and maintains BBB integrity. **(A, B)** The oxidative stress pathway proteins Keap1 and Nrf2 were detected by Western blot. **(C, D)** The ER stress pathway proteins ATF6 and GRP78 were detected by Western blot. **(E, F)** The BBB relative proteins ZO-1 and Claudin-5 were analyzed by Western blot. ^#^ *p* < 0.05, ^###^ *p* < 0.001, compared to control. *p < 0.05, **p < 0.01, ***p < 0.001, compared to H/R.

To further understand the protective mechanism of LQ on H/R-HBMECs, we first studied its oxidative stress pathway. Since Nrf2/Keap1 pathway plays a key role in oxidative stress, we investigated whether LQ could regulate the expression of these proteins. As shown in Figures 6A,B, LQ dramatically increased the levels of Nrf2 and decreased the expression of Keap1, indicating that Nrf2/Keap1 pathway involved LQ protects HBMECs from oxidative stress. Meanwhile, since ER stress is activated by ischemia-reperfusion, we investigated whether LQ could inhibit endoplasmic reticulum (ER) stress after H/R treatment. In our study, LQ reduced the levels of ATF6 and GRP78 in H/R-HBMECs, showing that the inhibitory effect of LQ on ER stress is involved in its protective mechanism, as shown in Figures 6C,D.

Furthermore, in order to clarify whether LQ protects HBMECs from H/R injury and can maintain the integrity of the BBB, we studied the expression of ZO-1 and Claudin-5 by LQ, and once the BBB is disrupted, the expression of both will be reduced. As shown in Figures 6E,F, ZO-1 and Claudin-5 expression decreased when subjected to H/R injury, indicating that BBB integrity was disrupted, and LQ could increase the expression of ZO-1 and Claudin-5 to maintain BBB integrity. Our results showed that LQ could inhibit oxidative stress and ER stress, protect HBMECs, and further maintain the integrity of BBB.

### iTRAQ Proteomics Analysis

#### Differentially Expression Proteins

**FIGURE 7:**
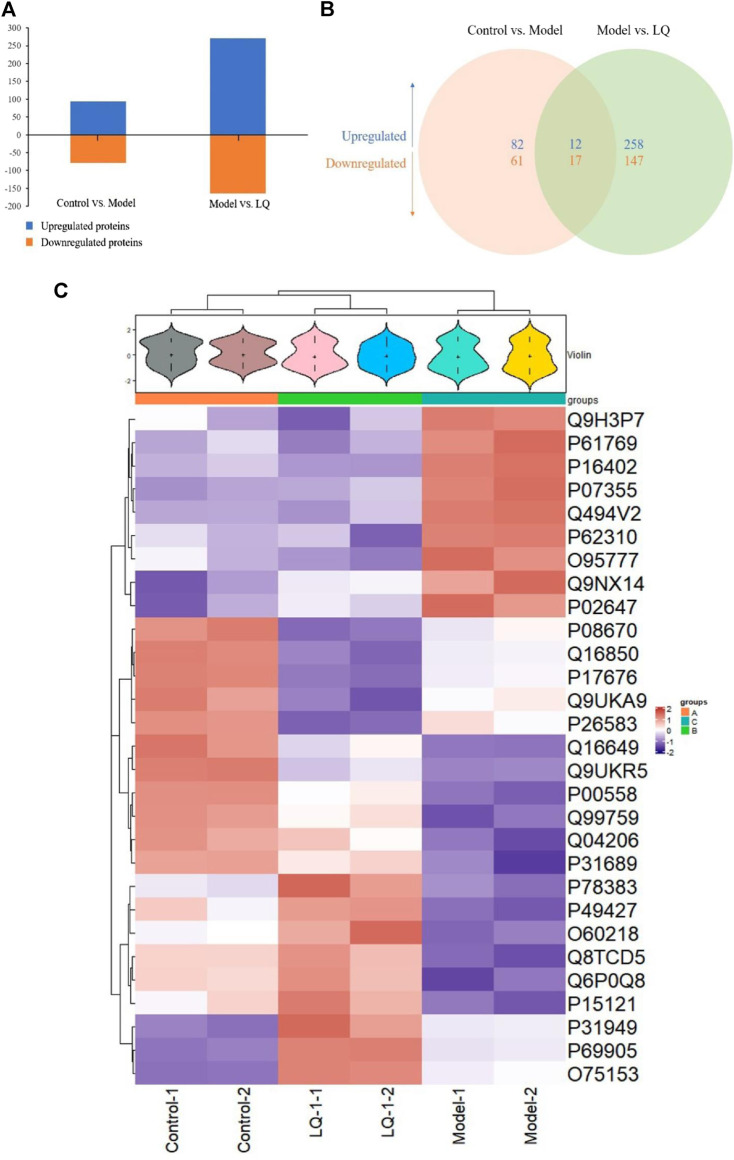
iTRAQ-bases quantification of the proteomes. **(A)** The number of up- and downregulated proteins in the two-comparison group. **(B)** Venn diagram of the distribution in each comparison group. **(C)** Heatmap of the 29 common differentially expressed proteins. Model: Hypoxia/reoxygenation; LQ: Liquiritin.

To examine the difference in H/R-HBMECs in the presence of LQ or not, we conducted the proteomics analysis by iTRAQ. Proteins exhibiting a *p*-value < 0.05 and a ratio fold change >1.2 or <0.83 were defined as differentially expressed proteins. In iTRAQ labeling, 172 proteins were identified as differentially expressed proteins between the control and model groups, among which 94 were upregulated and 78 were downregulated in the model group. There were 434 proteins identified as differentially expressed proteins between the model group and the LQ group, of which 270 were up-regulated and 164 were down regulated in the LQ group, as shown in Figure 7A. 29 of the same differentially expressed proteins were shared between the two groups, as shown in Figure 7B, Table1. The expression of these differentially expressed proteins was analyzed by hierarchical clustering as shown in Figure 7C.

**TABLE 1:** The common differentially expressed proteins.

Accession	Gene Name	Description
P07355	ANXA2	Annexin A2
P16402	H1-3	Histone H1.3
P31949	S100A11	Protein S100-A11
O75153	CLUH	Clustered mitochondria protein homolog
P69905	HBA1	Hemoglobin subunit alpha
O95777	LSM8	U6 snRNA-associated Sm-like protein LSm8
P61769	B2M	Beta-2-microglobulin
P02647	APOA1	Apolipoprotein A-I
P62310	LSM3	U6 snRNA-associated Sm-like protein LSm3
Q9NX14	NDUFB11	NADH dehydrogenase [ubiquinone] 1 beta subcomplex subunit 11, mitochondrial
Q9H3P7	ACBD3	Golgi resident protein GCP60
Q494V2	CFAP100	Cilia- and flagella-associated protein 100
P08670	VIM	Vimentin
P00558	PGK1	Phosphoglycerate kinase 1
P31689	DNAJA1	DnaJ homolog subfamily a member 1
P26583	HMGB2	High mobility group protein B2
Q9UKA9	PTBP2	Polypyrimidine tract-binding protein 2
P15121	AKR1B1	Aldo-keto reductase family 1 member B1
O60218	AKR1B10	Aldo-keto reductase family 1 member B10
Q16850	CYP51A1	Lanosterol 14-alpha demethylase
Q9UKR5	ERG28	Ergosterol biosynthetic protein 28
Q6P0Q8	MAST2	Microtubule-associated serine/threonine-protein kinase 2
P17676	CEBPB	CCAAT/enhancer-binding protein beta
P78383	SLC35B1	Solute carrier family 35 member B1
Q8TCD5	NT5C	5′ (3′)-deoxyribonucleotidase, cytosolic type
Q99759	MAP3K3	Mitogen-activated protein kinase kinase 3
Q04206	RELA	Transcription factor p65
Q16649	NFIL3	Nuclear factor interleukin-3-regulated protein
P49427	CDC34	Ubiquitin-conjugating enzyme E2 R1

#### GO Analysis

**FIGURE 8:**
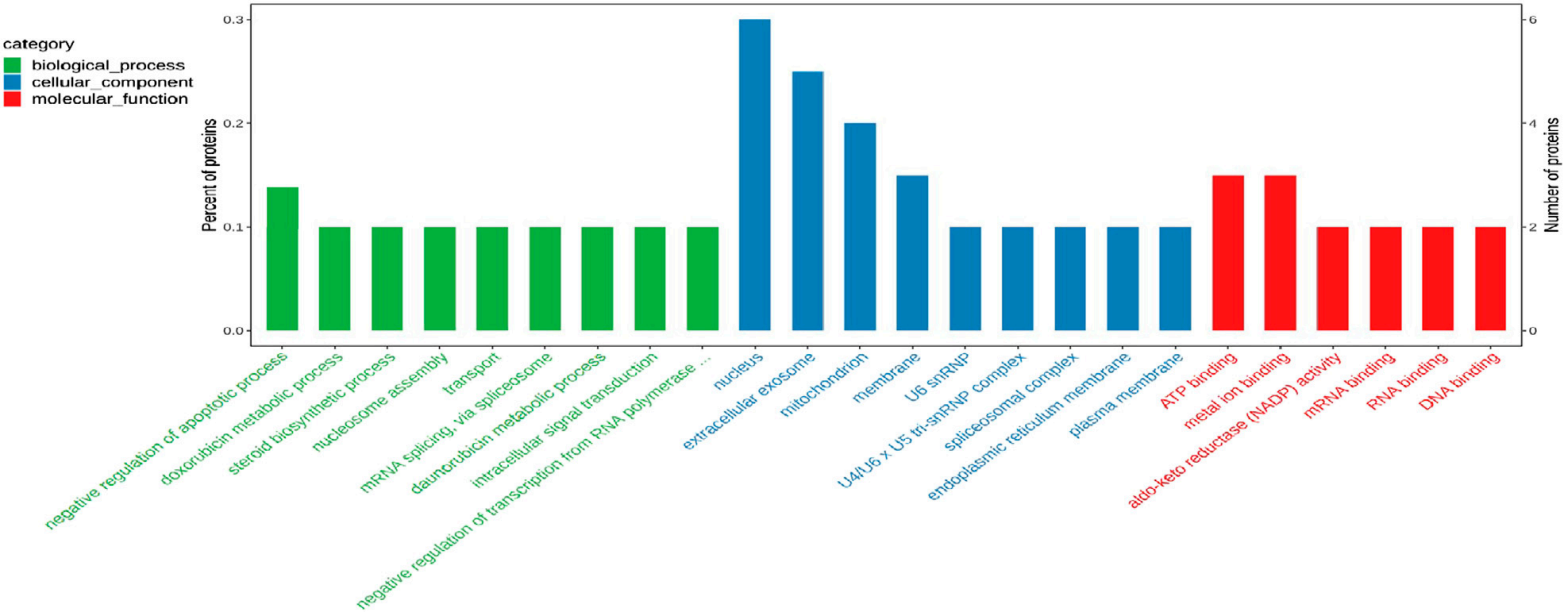
Gene ontology (GO) enrichment analysis of 29 common differentially expressed proteins.

To elucidate the biological significance of the 29 differentially expressed proteins, GO analysis was first performed on the differentially expressed proteins and then they were classified according to molecular function, biological process, and cellular component. The data of each category are shown in Figure 8. In the biological function category, the negative regulation of apoptosis process appeared significant enrichment. Under the cellular component, there was significant enrichment in the nucleus, cellular exosomes, mitochondria and membrane. In addition, ATP binding and metal ion binding are the most abundant in the category of molecular function.

Among the 29 differentially expressed proteins, five proteins were related to cerebrovascular diseases, including ANXA2, ApoA1, PGK1, CEBPB and MAP3K3. Among them, ANXA2 can be used to rescue cross endothelial cell density and BBB function after brain injury and CNS disease (Li et al., 2019). ApoA1 is reported to regulate the expression of apolipoprotein in brain and reduce atherosclerosis (Button et al., 2019; Contu et al., 2019). The increase of PGK1 can also reduce the formation of atherosclerosis (Zhang et al., 2020). CEBPB is involved in the regulation of brain injury and inflammation (Cortes-Canteli et al., 2008). MAP3K3 plays an important role in maintaining neurovascular integrity (Fisher et al., 2015). This may be the key protein for LQ to protect H/R-HBMECs. Future studies will verify the role of these proteins in LQ to protect H/R-HBMECs.

## Discussion

After ischemic stroke, I/R injury-mediated BBB rupture is associated with vascular leakage, circulating cells, and solute infiltration, leading to worsening edema and parenchymal damage (Granger 320 and Kvietys, 2015; Fu et al., 2015). Maintaining the integrity of the BBB may be a promising therapeutic strategy. Studies have shown that the protection of I/R-injured BMECs can reduce the increase of BBB permeability and cerebral infarction area after permanent occlusion of middle cerebral artery in rats caused by ischemic injury (Fang et al., 2016). Moreover, blocking the monolayer hyperosmolarity of HBMECs induced by inflammatory factors, loss of tight junction with surrounding cells and expression of adhesion molecules can effectively alleviate neurological deficits and ischemic injury in rats (Zhang et al., 2017). In this study, we used H/R to simulate ischemia-reperfusion *in vitro*, which is helpful to study the protective effect of LQ on HBMECs.

In this study, the safe and effective dose of LQ was screened out through the detection of cell viability. Subsequent studies were carried out under the premise of ensuring drug safety and non-toxicity. Promoting angiogenesis after restoring blood supply can protect cells against environmental stress including hypoxia (Li et al., 2017). This study was to systematically investigate the potential effects of LQ on angiogenesis using tubule formation assay and endothelial cell migration. Our results showed that LQ could effectively promote angiogenesis under H/R stimulation, such as tube formation and migration. Increasing evidence suggested that endothelial cell apoptosis may mediate brain dysfunction, suggesting that I/R-induced cerebrovascular lesions may involve microvascular endothelial cell apoptosis. Our study showed that LQ could reverse the decrease of mitochondrial membrane potential due to early apoptosis, thereby reducing apoptosis.

When the BBB is threatened, the expression of ICAM and VCAM increases, leading to the release of neurotoxic substances by neutrophils and monocytes, which in turn damage brain cells (Willam et al., 1999; Volcik et al., 2010). In this study, we found that LQ could reverse the elevation of ICAM-1 and VCAM-1, reduce their adhesion to surrounding toxic substances, and ultimately played a role in brain protection. In addition, after cerebral ischemia, cells will produce a large number of free radicals, such as ROS, excessive free radicals are the main mechanism of cell lipid peroxidation, which is one of the important causes of cell damage. At the same time, the activity of oxidases such as MDA will increase, which will destroy the balance of oxidation-antioxidation in the body, leading to severe damage of the blood-brain barrier and further worsen brain injury (Schreibelt et al., 2007). Our study found that LQ could decrease MDA expression, maintain oxidative balance and reduce brain injury. Besides, antioxidant enzymes such as SOD will also be reduced after ischemic events (Schreibelt et al., 2007). However, in our study, SOD level increased in H/R group, and the expression of it decreased after the treatment of LQ. We guessed that stress protection measures occur after cells were injured, forcing SOD level to rise, while LQ alleviated oxidative stress damage, thereby reducing SOD levels.

Nrf2 is an important transcription factor regulator and plays an important role in oxidative stress. Therefore, Nrf2 signaling is considered a therapeutic target for several human diseases (Taguchi et al., 2011). Keap1 is a negative regulator of Nrf2. When the body is subjected to oxidative stress, Keap1 decouples from Nrf2, and Nrf2 transfers into the nucleus. It combines with ARE, an antioxidant response element, and promotes the expression of antioxidants to enhance the antioxidant capacity of cells (Komatsu et al., 2010). When stimulated by oxidative stress, the Keap1/Nrf2 signaling pathway is activated, the body undergoes self-antioxidant response, and Nrf2 expression will elevated, but this is not sufficient to guarantee its anti-oxidative stress damage. Our study found that LQ could down-regulate the expression of Keap1, activate the downstream Nrf2/ARE antioxidant pathway, and protect cells from damage caused by H/R.

Meanwhile, ischemia and oxidative stress can lead to disorders in the folding of ER proteins, which can stimulate ER stress (Hetz, 2012). ER stress can be activated through three pathways: PKR-like ER kinase (PERK), inositol requiring enzyme 1 (IRE1), and the activating transcription factor-6 (ATF-6), which signal transduction proteins protect cells from ER stress under physiological conditions (Hetz et al., 2020). However, when the organism is threatened by the external environment, ER stress can induce apoptosis through these three pathways. And studies have shown that inhibiting ER stress can reduce brain injury after I/R injury (Nakka et al., 2010). Our study showed that H/R led to the activation of ER stress system, elevated expression of ATF6 and GRP78, and caused apoptosis. However, LQ could reduce the expression of both, inhibit ER stress, and then reduce apoptosis.

In addition, in order to find the possible targets of LQ, proteomic analysis was carried out. 29 common differential proteins were detected among the control group, H/R group and LQ group. Among them, ANXA2, ApoA1, PGK1, CEBPB and MAP3K3 were related to atherosclerosis and cerebral inflammation. Therefore, future studies will verify the role of these proteins in the protection of H/R-HBMECs by LQ.

However, there are still many limitations in this experiment. In our study, for BBB integrity experiment, we only detected the expression of ZO-1 and Claudin-5, which are tight junction proteins and play an important role in BBB skeleton maintenance. However, in order to describe the integrity of BBB in many aspects, trans epithelial electric resistance also needs to measure the permeability. Secondly, for proteomic analysis, only five common differential proteins were described in this study, which may be the target proteins of LQ protecting H/R-HBMECs, but still need further experiments to be verified.

In conclusion, we investigated the protective effects of LQ on H/R-HBMECs. Our study showed that LQ could significantly improve the damage of HBMECs caused by H/R, which manifested as: enhancing cell viability, cell migration and reducing cell apoptosis. We further explored the mechanism of action of LQ and found that LQ could reduce ROS and MDA levels, as well as SOD expression, which may be related to its activation of Keap1/Nrf2 antioxidant pathway. Not only that, our study also demonstrated for the first time that LQ can reduce apoptosis by inhibiting ER stress, which provides the experimental basis for the compound ratio in the future. In the future, we should further study its targets in order to provide new treatment ideas for stroke.

## Data Availability

The data presented in the study are deposited in the iProX repository, accession number is LMT902.
